# Efflux, Signaling and Warfare in a Polymicrobial World

**DOI:** 10.3390/antibiotics12040731

**Published:** 2023-04-08

**Authors:** Ay’sha Moore-Machacek, Antje Gloe, Niall O’Leary, F. Jerry Reen

**Affiliations:** 1 School of Microbiology, University College Cork, T12 K8AF Cork, Ireland; 2 Institute for Pharmaceutical Microbiology, University of Bonn, D-53113 Bonn, Germany; 3 Synthesis and Solid-State Pharmaceutical Centre, University College Cork, T12 K8AF Cork, Ireland

**Keywords:** efflux, polymicrobial infections, biofilms, antimicrobial resistance, microbiome, effluxome, symbiosis

## Abstract

The discovery void of antimicrobial development has occurred at a time when the world has seen a rapid emergence and spread of antimicrobial resistance, the ‘perfect storm’ as it has often been described. While the discovery and development of new antibiotics has continued in the research sphere, the pipeline to clinic has largely been fed by derivatives of existing classes of antibiotics, each prone to pre-existing resistance mechanisms. A novel approach to infection management has come from the ecological perspective whereby microbial networks and evolved communities already possess small molecular capabilities for pathogen control. The spatiotemporal nature of microbial interactions is such that mutualism and parasitism are often two ends of the same stick. Small molecule efflux inhibitors can directly target antibiotic efflux, a primary resistance mechanism adopted by many species of bacteria and fungi. However, a much broader anti-infective capability resides within the action of these inhibitors, borne from the role of efflux in key physiological and virulence processes, including biofilm formation, toxin efflux, and stress management. Understanding how these behaviors manifest within complex polymicrobial communities is key to unlocking the full potential of the advanced repertoires of efflux inhibitors.

## 1. Introduction

Efflux pumps are ancient transport systems ubiquitous across all the domains of life. Perhaps best known for their role in antimicrobial resistance (AMR), there is a growing awareness that efflux pumps play important, but poorly understood ecological and physiological roles in natural ecosystems. This is demonstrated by their evolutionary conserved nature (at DNA and protein sequence levels) [1]. Efflux pumps are generally chromosomally encoded and largely considered part of the “core genome”, although many appear not to be essential for growth [2]. This could either be due to the functional redundancy of efflux pump systems or may simply reflect the non-essential nature of these systems under the specific testing conditions. The absence of defined natural substrates for many classes of efflux system hampers the extent to which their physiological role can be understood. Furthermore, in some cases efflux pump genes can also be acquired via horizontal gene transfer, increasing the complexity of efflux potential available within communities [1].

The current Post-Antibiotic Era we are facing describes the rise in AMR and even more worryingly, multi-drug resistance (MDR) mechanisms in pathogens and the subsequent lack of novel antibiotic discovery [3]. “Common infections”, such as urinary tract infections (UTIs) and sexually transmitted infections (STIs) that are currently manageable via antibiotic administration will eventually no longer be treatable, leading to a rise in morbidity and mortality rates. Currently, many chronic infections are being treated with “last resort” antibiotics to prolong the patient’s life, despite cytotoxicity issues, such as treating sepsis with carbapenems [4]. Currently, AMR and MDR infections are responsible for 1.2 million deaths annually. The WHO has listed five pathogens, the ‘ESKAPE’ pathogens, as of most concern regarding AMR and lack of effective treatments. ESKAPE is an acronym for *Enterococcus faecium*, *Staphylococcus aureus*, *Klebsiella pneumoniae*, *Acinetobacter baumannii*, *Pseudomonas aeruginosa*, *Enterobacter* spp. [5]. *S. aureus*, particularly methicillin-resistant *S. aureus* (MRSA) infections currently have a 60% increased mortality rate compared to drug-sensitive infections; and *K. pneumoniae* resistance to carbapenems is increasing worldwide. This is an overall global trend with microbial pathogens [6]. Though there are multiple reasons behind this AMR and MDR trend, such as natural selection and anthropogenic activities such as over-use/improper antibiotic usage, resistance mechanisms via efflux pumps are a leading contributing cause [7].

Efflux pumps can be divided into superfamilies and classes (Table 1) according to the (i) number of components (single or multiple), (ii) number of transmembrane-spanning regions, (iii) energy source, and (iv) sequence similarity [8]. These major families are the adenosine triphosphate (ATP)-binding cassette (ABC) superfamily; the resistance-nodulation division (RND) family; the major facilitator superfamily (MFS); the small multidrug resistance (SMR) family; the multidrug and toxic compound extrusion (MATE) family; the proteobacterial antimicrobial compound efflux (PACE) family; and the *p*-aminobenzoyl-glutamate transporter (AbgT) family [2]. Efflux pumps in Gram-negative bacteria can be further classified on whether they span both membranes (tripartite systems) or just span the inner membrane (single component efflux pumps) [2]. These single-component efflux pumps work in conjunction with tripartite systems, where they transport their substrates to the periplasmic space, followed by extracellular extrusion via tripartite systems. Genes encoding these functionally and structurally diverse efflux pump systems are widespread in the microbial world with significant repertoires of most efflux pump families present in each species or strain [1]. With respect to the community level functioning of efflux systems, the prevalence of efflux systems encoded in genome may impact on strain dynamism, virulence, defense against host-derived molecules, and biofilm formation, though for the most part the specific signals involved remain to be characterized [9,10].

The number of efflux pumps/multi-drug efflux pumps in a genome of a particular species is related to the overall genome size, which correlates with the bacteria’s ecology and the environment(s) it inhabits. Environmental, free-living bacteria tend to have large genomes and, thus, code for a large variety of efflux pump types to enable them to survive the various harsh conditions they are exposed to [19]. In contrast, due to reductive evolution, pathogens and endosymbionts typically possess a reduced genome size and thus, less efflux pumps in their arsenal [19]. Pathogenic clinical isolates make up for this reduced variety of efflux pump systems by overexpressing their efflux pump arsenal in response to various host triggers [20]. *Pseudomonas aeruginosa*, a Gram-negative nosocomial pathogen with a large genome size, benefits from the overexpression of the MexEF-OprN efflux pump without conferring a fitness cost [20]. This is perhaps an anomaly, rather than the status quo, as metabolic rewiring of its aerobic respiratory chain (via increased expression of anaerobic nitrate respiratory chain under aerobic conditions) was the trade-off for the lack of fitness cost. Thus, oxygen gradients are important inducers/repressors of efflux pump expression, perhaps as means of extruding toxic anaerobic waste build-up [21]. Pathogens also make up for their reduced variety of encoded efflux pumps by encoding multi-drug resistant (MDR) efflux pumps. In fact, the expression of a single efflux pump can confer MDR due to substrate promiscuity. These resistant species are then selected during infection and treatment with antibiotics and are both beneficial to these MDR efflux pump producers and non-producers in the community [22].

**Figure 1:**
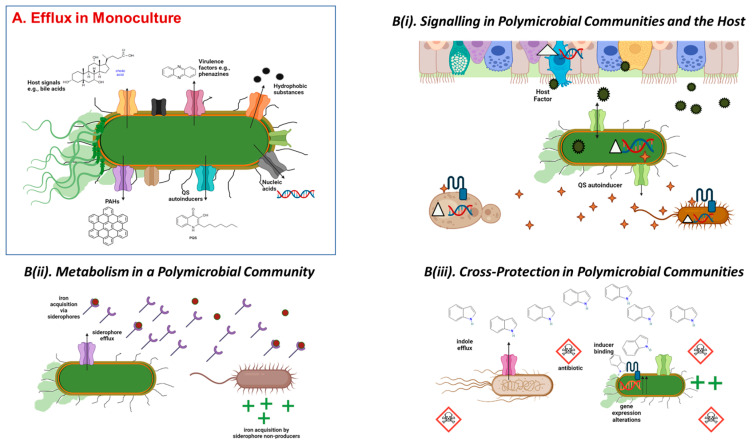
(**A**) Efflux in monoculture has been studied extensively, with a broad spectrum of systems for which substrates have been profiled and characterized. Perhaps the best studied of these have been antibiotics in the context of AMR mediated through efflux of specific classes of intracellular targeting drugs. (**B**) Efflux in a polymicrobial context. (**i**) Host signaling and interspecies/interkingdom communication systems in bacteria, yeast, and fungi, create a complex networked environment where efflux systems and their inhibitors can have a significant input into the dynamic outcomes of intervention. Furthermore, affinities of EPI’s for efflux pumps may vary at the species level, with potential for off-species effects targeting as yet uncharacterized community members, thus insulating the intended target from the full dose of the intervention. (**ii**) Inhibiting the metabolic role of efflux systems in expulsion of metabolites and scavenging factors such as siderophores could have important consequences within polymicrobial communities where, e.g., iron acquisition by siderophore non-producers is affected through inhibition of symbiotic partners. (**iii**) The cross-protection afforded to some species of bacteria can also be lost through efflux inhibition whereby, e.g., protection arising from recognition of indole efflux by *Escherichia coli* is lost to *Pseudomonas putida*. Created using Biorender.com.

Understanding the physiological basis of efflux systems and how they function at the community level is critical to deciphering the dynamic networks that underpin polymicrobial communities or microbiomes. Efflux does not occur in a singular context, but rather within complex microbial consortia, often encased within a biofilm structure. It follows therefore that efflux from one organism may often necessitate or facilitate uptake by a neighboring cell or species [8,23]. Where this is the case, the impact of efflux inhibition may extend beyond pathogen inhibition, and the consequences of this need to be understood (Figure 1). Some key physiological roles of efflux pumps in bacteria will be discussed, such as homeostasis and waste extrusion, cell-cell signaling, the role of outer membrane vesicles (OMVs), host colonization and AMR, with a focus on exploring the role of efflux systems in the context of mixed microbial communities.

## 2. Ecological Role of Efflux Pumps

### 2.1. Efflux Pumps, Homeostasis and Stress Management

Bacterial cells are tightly regulated to prevent toxic metabolite (waste) accumulation. Efflux pumps are most commonly known for their role in extruding exogenous, toxic compounds such as antibiotics from bacterial or fungal cytoplasm into the extracellular environment. However, the physiological role of efflux in host homeostasis and waste extrusion is less appreciated and not as well understood. Through efflux pump functional redundancy and substrate promiscuity, one efflux pump can extrude a variety of structurally similar compounds, such as, e.g., bacterial secondary metabolites [8], in some cases enabling prevention of toxic primary metabolite/(waste) accumulation intracellularly. The ability to extrude exogenous, potentially harmful substrates is considered (though not yet universally shown to be) a potential key natural role for efflux systems. For example, bacteria can expel a wide variety of substrates that would otherwise be toxic to the cell, such as biocides, heavy metals, organic pollutants, host signals (bile in mammals, plant exudates) antimicrobials (produced by other microbes in the vicinity or administered clinically), etc. [2].

Iron-scavenging siderophores are known to be extruded by ABC and RND-type efflux pumps, allowing access to bio-available iron in iron-limited environments, such as in human infections [2]. Siderophore production confers a fitness advantage to the producer, as well as to neighboring cells with the relevant uptake system [24]. Siderophore extrusion also confers a secondary protective effect against reactive oxygen species (ROS) and reactive nitrogen species (RNS), something in which both the MATE and RND efflux pump families are involved [2]. Guanidine is a common component of the nucleic acid base, guanine, and the amino acid, arginine. At high intracellular levels, guanidine disrupts cell membrane permeability and can be toxic. Therefore, this endogenous substrate is also extruded via SMR-type efflux pumps to maintain cell homeostasis. Though endogenously produced sugars, amino acids, etc., typically possess their own specific transporter systems, efflux pumps are also commonly involved as a means of cellular detoxification. Membrane fatty acids can be extruded via efflux pumps (of the RND family, in particular) because of turnover or damage [2]. Though less mechanistically understood, polyamines are also expelled via SMR, MFS and RND-type efflux pumps. Polyamines have been implicated in various physiological systems, such as the induction of acidic stress response systems and conferring protection against ROS/RNS. Overexpression of the Blt transporter in *Bacillus subtilis* was reported to confer a multidrug-resistance phenotype in addition to effluxing large amounts of spermidine into the medium [25]. Unsurprisingly, therefore, this system has been the focus of some novel inhibitor design [26]. Short chain diamines have been described in *Acinetobacter baumannii* as physiological substrates for the proteobacterial antimicrobial compound efflux (PACE) family of efflux systems [27]. Similar to polyamines, diamines play an important role in bacterial physiology and pathogenesis and can themselves be developed as potent antimicrobial scaffolds [28]. 

Efflux pumps are critical in maintaining cytoplasmic pH homeostasis, which in turn enables the functionality of efflux pumps through maintaining proton motive force (PMF) and the function of key metabolic enzymes. Therefore, when faced with extreme pH stresses (e.g., in the mammalian gut, in acidic lakes, alkaline lakes, etc.), the expression of efflux pumps and other systems are modulated to ensure membrane stability, permeability and host homeostasis. In fact, for species inhabiting harsh environments, efflux pumps underpin their extreme pH tolerance and even acid resistance, in some cases [2].

### 2.2. Efflux Pumps and Signalling

While endogenous signaling molecules, such as quorum sensing (QS) autoinducers, are typically diffusible, transport to the extracellular milieu is also known to occur via efflux pumps [2]. QS is the phenomenon of cell-density-related gene expression. QS systems are cell-cell communication systems on the species level, interspecies level and even on the interkingdom level [29,30,31]. QS is a community-coordinated phenomenon, involved in regulating key virulence factors, such as biofilm formation, swarming motility, and extracellular protein production [32]. Many QS systems are controlled by two-component systems (TCSs)/multikinase networks, responding to various environmental inputs and regulating the expression of many genes in this network. Numerous efflux pump systems have themselves been linked to extracellular autoinducer transport [33], as seen with the MexAB-OprM, MexCD-OprJ, MexEF-OprN, and MexGHI-OpmD systems involved in the modulation of the *P. aeruginosa* QS response. Mutation or overproduction of the genes encoding these efflux systems has reportedly led to alterations in QS signaling and the accumulation of signals in the extracellular environment [34,35]. However, the redundancy and hierarchy in these key signal transduction networks are such that the impaired production of one signal can lead to the attenuation of functions attributed to another [36]. In several cases, a shift in efflux patterns for key intermediates of the *Pseudomonas* Quinolone Signal (PQS) signaling system in *P. aeruginosa* occurred through overproduction of the MexEF-OprN efflux pump [34,35,36], previously described as a 3-oxo-C12-HSL LasIR related system [37]. While a lack of significant impact on competition was reported for MexEF-OprN overproduction when compared to wild-type strains [35], this single species analysis may hide a more complex role at the interspecies or even interkingdom level given the emerging role for this signaling system in the microbe-microbe and microbe-host interaction [31,38,39]. Efflux pumps may play a key role in mediating interspecies and interkingdom communication, as well as extruding “foreign” autoinducer molecules from the cell [33]. Potentially, this could act as a defense mechanism against other competing, neighboring bacteria present in the polymicrobial community. 

The biofilm formation is dependent on QS and is a community-coordinated behavior, it is perhaps unsurprising that efflux pumps reportedly also play a key role—mediating the various regulatory and communication systems required to maintain and finally disperse the biofilm [33]. A positive correlation between efflux pump expression and biofilm formation has been reported in several studies [33], with AcrAB-TolC of *E. coli*, MexAB-OprM of *P. aeruginosa*, AdeFGH of *A. baumannii* and AcrD of *S. enterica*, playing important roles [40]. This is an important consideration for strategies that seek to target efflux systems, indicating that the primary target of such approaches will be functionally relevant in what is understood to be the principle lifestyle adopted by microbes in environmental and host-associated ecosystems [41]. 

### 2.3. Efflux Pumps, Antibiotic Stress, and Outer Membrane Vesicles

Outer membrane vesicles (OMVs) are proteolipid nanostructures of endocytic origin that are naturally secreted during normal growth from the outer membrane of Gram-negative bacteria [42,43]. OMVs are involved in ferrying various substrates, including virulence factors such as toxins, outside of the cell. OMVs are one of several primary mechanisms of microbial signal transduction [44], and they play crucial roles in microbe-microbe and microbe-host interactions [42]. OMVs can act as delivery systems between other microbes or the host [44]. There is some evidence to suggest that OMV fusion can program naïve cells to more rapidly adapt to harsh environments [45,46]. The synthesis of OMVs and its cargo varies depending on environmental cues, such as growth phase, mode of growth, oxygen availability, etc., and significant heterogeneity exists with respect to their composition, even at the single species level [47]. Stressful conditions dramatically alter OMV composition and cargo, such as equipping OMVs with multidrug efflux capabilities or degradative abilities for any potentially harmful substances in the extracellular environment [43]. Huang and co-workers describe a novel antibiotic efflux phenomenon mediated by *A. baumannii* OMVs, which resulted in enhanced drug resistance [44]. When stressed with antibiotics, the expression of the AdeAB and AcrB-mediated efflux pump was dramatically altered [44]. This altered expression, and subsequent activation of efflux pumps under antibiotic stress was shown to result in encapsulation of large amounts of intracellular components into OMVs. Coupling with the low expression of the outer membrane proteins prevents the antibiotics that have been packaged in the OMVs from being released again. Some key metabolic-related proteins (involved in carboxylic acid, small-molecule, organic acid, etc., pathways) and small molecules involved in key signaling pathways (involved in small molecule binding, cofactor binding, catalytic activity, etc.) were also encapsulated into these OMVs and extruded from the cell. Though the exact mechanism of action has yet to be established, it seems OMVs and efflux pumps may co-operate to provide a crucial modality for microbial fitness and competitiveness in the hostile environments microbes inhabit. This novel mechanism was itself utilized to develop an innovative antibiotic loading strategy for control of pathogen infection in mice, highlighting again the intrinsic value in understanding the physiological basis of microbe-host interactions [44]. In *Neisseria meningitidis*, the opposite may be seen whereby the multidrug efflux pump channel protein MtrE was less abundant in OMVs than in the outer membrane [47]. Therefore, further research is needed to appreciate the complexity of the efflux-OMV relationship, particularly in the context of the emerging role OMVs with respect to microbe-microbe symbiosis [48] and microbe-host immunomodulatory interactions [49].

### 2.4. Efflux Pumps and Host Colonisation

Efflux pumps play a significant role in efficient host invasion/colonization and subsequent dissemination [19]. Efflux pumps facilitate microbial resistance to host defense compounds or cellular mechanisms, such as salicylic acid and phenolic acids in plants, or bile acids and phagocytosis in animal hosts [15,19]. Though challenging to overcome from a host perspective in the case of pathogenic microorganisms, this efflux pump-mediated resistance to the host can underpin symbiotic relationships, as seen in the case of rhizobia colonizing legume plants or commensal bacterial colonization of the mammalian gut [15]. The converse is true in the case of bacteria-phage coevolution, whereby efflux pumps can be an Achilles heel, acting as receptor binding proteins (RBPs) for phage invasion [50]. While efflux pumps display substrate promiscuity, bacteria-phage interactions tend to be much more specific. The same efflux pumps present in different species can act as RBPs for various phage, as seen with TolC and various *Salmonella* serovars. However, this is not always the case and efflux pump sequence similarities ultimately dictate bacteriophage adsorption to its RBP [50]. 

An interesting relationship between efflux and host colonization can be seen in the hypoxic environment that exists during infection and chronic colonization [51]. There is increasing evidence of a distinct and important contributing role for hypoxia in the dysfunction of the airway epithelium and in the responses of both innate immunity and of respiratory pathogens [52]. Micro-anaerobic environments, such as those that exist in the lungs of patients with Cystic Fibrosis (CF), are known to promote multi-drug resistance and the increased MIC of antibiotics through alterations in signaling, efflux, and metabolism [53,54]. In the host, the Hypoxia-Inducible Factor (HIF)-1 is involved in multi-drug resistance, controlling the MDR1 P-glycoprotein (P-gp) that belongs to a family of ATP-binding (ABC) cassette transporters [55]. ATP synthesis and thus microbial metabolism and oxygen availability is critical here for active efflux to occur. Other efflux pumps operating via proton motive force (PMF) require energy flow through the electron transport chain and the presence of an energized membrane [56]. Hypoxic control of efflux in the host also extends to key physiological processes such as sterol synthesis [57], lactate secretion and glycolytic efflux [58], and potassium transport [59]. At the same time, HIF-1 plays an important role in moderating the host’s response to infection, recognizing pathogen colonization, while also mediating an effective immune response [60]. Bile acids, a human hormone-like factor that has been recently identified in the lungs of patients with CF and other respiratory conditions [61,62], are known to destabilize the HIF-1α subunit of HIF-1, resulting in altered expression of downstream regulatory components of the inflammatory and immune responses [63,64]. At the same time, bile acids promote production of the *P. aeruginosa* alkyl-4-quinolone (AQ) signaling system which itself is able to destabilize HIF-1α [38,65], and can be effluxed through the cell via the MexEF-OprN system [66]. While the role of efflux in this complex interplay remains to be fully determined, the dual targeting of such an intrinsic system suggests further research is warranted. 

## 3. Efflux Pump Regulation

Regulation of efflux pumps plays an important role in moderating the appropriate physiological response and may itself represent a feasible inhibitory target given the signal or co-inducer responsive nature of many of the efflux regulatory systems [20,67,68]. MDR efflux pump components are typically encoded in an operon to aid efficient and complete assembly, and are regulated via TCSs/multikinase networks [69]. The core components of typical TCSs include a membrane-bound sensor histidine kinase, one or multiple signal transduction components and a response regulator (transcription factor), where a phosphorelay occurs to alter the expression levels of many genes. This is not a simple on/off switch, instead depending on the levels of the phosphorylated response regulator. This enables the microbe to respond to various environmental cues in real time, as well as influencing other regulatory circuits. When the inducer(s) for the efflux pumps are not present, the pumps are significantly downregulated, with minimal basal expression [70]. Numerous transcriptional regulators—both global and local—keep the various encoded efflux pumps tightly regulated. Efflux pumps and QS are intrinsically linked and are capable of influencing each other, where the upregulation of one system leads to the downregulation of the other system [33].

Efflux pump components are regulated at numerous different levels to fine tune responses to ever-changing environments and to avoid unnecessary energy costs. Such regulation points are at the transcriptional, post-transcriptional, translational, and post-translational levels [69]. Transcription and post-transcription factors, such as RNA regulatory switches (both cis- and trans-acting RNAs) play critical roles in efflux pump expression levels [69]. Small proteins governing the expression of membrane channels (including components of efflux pumps) are also key here. More recently, a study using dynamic Boolean modeling has suggested that ATP variability and energy supply may control efflux pumps, with model bacteria developing heterogeneous pulses of efflux pump gene expression, which contribute to a sustained sub-population with increased efflux expression activity [71]. The regulation of these systems and their components is discussed in greater detail below and summarized Table 1.

### 3.1. RND Efflux Pumps in Pseudomonas aeruginosa and Their Regulation

The RND family are one of the largest efflux pumps known and are typically tripartite in nature. The RND efflux pumps in *P. aeruginosa* are one of the most extensively studied efflux pump families. For some RND efflux pumps, a partner outer membrane factor (OMF) protein is also encoded in the same operon, allowing substrates to pass out of the Gram-negative outer membrane. If this OMF is not present in the same operon as an efflux pump, the efflux pumps can assemble with a more universal OMF [67]. Up to 12 RND efflux pumps have been identified in the *P. aeruginosa* PA01 genome, including MexA, MexB, MexX, MexY, MexJ, and MexK [13,67,72,73,74]. Each has different substrate specificities but equally also exhibit distinct substrate promiscuities. MexAB-OprM differs from the other RND pumps as it is constitutively expressed in *P. aeruginosa* and displays wider substrate specificities than the other RND efflux pumps [13]. The MexGHI-OpmD efflux pump is particularly interesting in light of its apparent role in transporting phenazines, leading to the modulation of the gene expression and biofilm development in *P. aeruginosa* [74]. Phenazines have been shown to play a significant role in host colonization and competition within mixed microbial populations and have also been shown to drive diversification within subpopulations of *P. aeruginosa* [75,76,77]. The interconnection of AMR with strain diversification and the host interaction serves to highlight the complexity of targeting efflux within a living system. Of course, it also suggests there would be significant potential to modulate populations based on their behavior, rather than simply viewing EPIs as antibiotic adjuncts.

Though PA01 possesses ~130 different TCS, only 5 are known to be involved in the regulation of RND efflux pumps. These belong to the OmpR/PhoB family with the exception of RocS2-RocA, which is part of the CheY system for chemotaxis [67]. Other efflux pump regulators fall into the “one component system” (OCS) class, which are generally composed of a DNA-binding domain and a sensory domain. This class of regulators are involved in most crucial signaling events in bacteria and can act as both (local and global) activators and repressors depending on the location of their binding site. Examples of such regulators in this family involved in efflux pump regulation in *P. aeruginosa* are LysR, TetR and MarR. LysR-type transcriptional regulators (LTTRs) are the most abundant type of transcription factors (TF) in bacteria and are both structurally and functionally conserved [78]. Despite this, LTTRs are involved in the regulation of diverse gene sets, e.g., virulence, QS, and metabolism [78]. One of the most studied LTTRs in *P. aeruginosa* is MexT, a local regulator of the MexEF-OprN operon and a global regulator of virulence and pathogenesis [79,80]. Many of the RND efflux regulators in *P. aeruginosa* belong to the TetR family, so named for its discovery in relation to tetracycline resistance [67]. This family is involved in providing antibiotic resistance via efflux pumps, as well as regulating genes involved in the export of small molecules (perhaps for signaling purposes) [81]. The MarR (Multiple Antibiotic Resistance Regulator) family usually act as repressors of the diverse array of genes under their control, mainly those involved in responding to environmental factors and stressors such as nutrient availability, pH, temperature, oxidative stress, and toxic substances [82]. The MarR regulator MexR is involved in activating the MexAB-OprM operon in *P. aeruginosa*, with some evidence of a role for the AraC family in efflux regulation also reported in some species [83].

Table 2 summarizes the known RND two-component system and one-component system regulators in *P. aeruginosa*, with some of the known inducers, the efflux pump(s) affected and the resulting phenotype. It should be noted that many of the inducers outside of antibiotics are unknown, as efflux pumps are primarily studied due to their role in AMR and not for their ecological role. 

### 3.2. MFS Efflux Pumps in Staphylococcus aureus and Their Regulation

More than 15 efflux pumps systems have been described in S. aureus thus far [84], perhaps the best studied of these being the NorA efflux pump, a member of the MFS family [85]. The MFS family is the largest and most diverse membrane transporter, found across all the domains of life and it confers the most prominent resistance phenotypes compared to other efflux pump families [84]. 

MgrA (multiple gene regulator) is a global regulator and governs many of the MFS efflux pumps in *S. aureus*. MgrA is a MarR transcriptional regulator, whereby phosphorylation of MgrA allows access to the *NorA* promoter and, thus, transcription [86]. Both MgrA and NorR act as activators of the *NorA* gene, leading to drug extrusion, altered cell surface properties [87] and increased uptake into epithelial cells [88]. Further regulatory control of NorA occurs via NorG, a GntR (gluconate regulatory)-like protein family; activation occurs via Fur (ferric uptake regulator) as well as via the ArlR-ArlS TCS [86]. MgrA also regulates other ‘Nor efflux pumps’, such as NorB and NorC, normally via downregulation/repression [84,86]. However, under harsh conditions, such as in the host and during biofilm growth, where low pH and reduced oxygen levels is common, NorB is upregulated [89]. Further regulatory control is enacted via NorG activating NorB and repressing NorC [86]. NorD is not governed by MgrA, instead, it is downregulated by Fur in response to iron-limited conditions, such as in the host [19,86].

Another critical MFS efflux pump is Tet38, aiding in host cell colonization and invasion, and subsequent toxic compound extrusion [88]. MgrA, along with TetR21, downregulate Tet38 expression [88]. MgrA’s regulation of Tet38 is indirect, as only TetR21 binds directly to the promoter of *Tet38* [19]. Upon the sensing of various host fatty acids or upon *P. aeruginosa* co-infection, both NorB and Tet38 are upregulated via MgrA repression [88]. This can ultimately lead to the establishment of a chronic infection in the lungs of people with Cystic Fibrosis (CF) and in abscesses. TetA(K) is a plasmid-encoded MFS efflux pump involved in drug extrusion and Na+/K+ uptake [86]. This efflux pump protects the producer from harsh conditions, such as alkali stress, Na+ stress and lack of K+ [86], with putative substrates for this efflux pump, including fatty acids. 

QacA is one of the earliest identified MDR efflux pumps and is a member of the MFS family [19]. Since then, QacB has also been identified and characterized [86]. Like NorD, QacA/B are not governed by MgrA—instead they are downregulated by QacR, a TetR repressor [19,86]. QacA/B multidrug efflux expression is induced upon substrate binding to QacR, thus releasing it from the promoter and allowing transcription. Such substrates include various antimicrobials, plant compounds (alkaloids in particular), and host fatty acids [19]. However, some additional QacA/B substrates do not bind QacR, in which case QacR remains bound to the promoter. Basal expression of QacA/B overcomes this resting inhibition, with QacR known as a weak repressor [86]. 

Other MFS multidrug efflux pumps found in *S. aureus* include Mef(A), LmrS, and SdrM [86]. Little is known regarding the various regulators and substrates of these lesser-known efflux pumps—with the exception of SdrM, which is downregulated by the global regulator, MgrA [86]. Collectively, these efflux pumps are attractive targets for efflux pump inhibitors (EPIs), with the Nor efflux pumps in particular displaying a broad range of substrate promiscuity [84]. Alternative targets include MgrA, the global regulator for many of the MFS MDR efflux pumps in the clinically prevalent *S. aureus* [84]. However, further study is needed to better understand the various efflux pumps and their regulation, such that the looming AMR challenge can more effectively be addressed. This is especially true in polymicrobial community settings.

### 3.3. Cell-Cell Communication, Sensing the Qourum, and Efflux Regulation

Perhaps unsurprisingly given the influence efflux has on the transport and dissemination of quorum sensing signals and their precursors, QS has itself been shown to play a significant role in controlling efflux in a range of microbial species. In *E. coli*, the quorum sensing transcription factor SdiA was shown to up-regulate *acrAB* gene expression [90,91]. Similarly, transcriptional control of the *acrAB* operon by the QS transcriptional regulator AnoR has been reported in *A. nosocomialis* [92]. The AcrAB efflux system is involved in the extrusion of natural signal molecules, and has underpinned the hypothesis that synthetic drug export by AcrAB may resemble the repertoire of signaling molecules encountered in natural ecosystems [91]. This of course complicates the association whereby the loss of QS signals through functional studies may mimic efflux inhibition in the cell, resulting in a feedback loop of regulation. In the case of *P. aeruginosa*, the MexAB-OprM RND efflux system selectively interacts with bacterial signaling molecules, enabling binding of 3-oxo-C12-HSL but limiting the accessibility of non-cognate acyl-HSLs to the LasR receptor [93]. This relationship suggests a complex interplay between QS and efflux, though further work is needed to establish the hierarchical dynamics of this.

## 4. The Microbial Ecology of Efflux Inhibition and Sources for Exploration

### 4.1. On the Ecological Basis of Efflux Inhibition

The ecological context of efflux inhibition is an important dimension in understanding the functionality of what are typically viewed as important antibiotic-enhancers [94]. Efflux inhibition in a natural sense can impact on cell-cell communication, waste extrusion, QS, vesicle production and other functionalities that are core to microbial physiology. It follows, therefore, that apart from disrupting classical antibiotic efflux, an important role for efflux inhibition may lie in the anti-infective space, rather than simply focusing on growth inhibition through enhancement of antibiotic activity. While discussed below in the context of polymicrobial communities, at its simplest, the ability to block cell-cell communication, and by extension, multi-cellular behavior could suppress key virulence phenotypes such as biofilm formation, swarming motility, and toxin production [31,95,96]. This anti-infective approach has seen significant growth in research in recent years, particularly with regard to the action of small molecules that target quorum sensing receptors and two component systems. Efflux inhibitors should also be considered as potential anti-infective compounds where suppression of signaling and communication could offer an innovative strategy for control of what are often opportunistic pathogens. This form of pathogen control does not carry the same risk with respect to resistance, though recent studies suggest a fitness cost may still arise [97].

While the majority of studies in efflux inhibition have focused on bacterial systems, targeting efflux pumps to overcome antifungal drug resistance in eukaryotes has also shown some promise. Chemo-sensitizing *Candida albicans* to azole challenge has been a principal research focus, and some advances have been made in re-establishing the effectiveness of conventional anti-fungal compounds against resistant strains [98,99,100]. Such approaches are also likely to significantly impact the bacterial dynamics with a host ecosystem, whereby co-evolution of micro- and myco-biomes establish intricate networks that are quite distinct from the mono-species behaviors of laboratory isolates. 

### 4.2. Plants as a Source for Efflux Inhibitors

The search for efflux pump inhibitors (EPIs) is a challenging task as cytotoxicity towards human cells is a major factor in the development of EPIs for clinical use. Secondary metabolites with potential efflux pump inhibitory activity can be grouped into three categories: terpenes, phenolic compounds, and alkaloids. Geraniol (monoterpenoid) and farnesol (acrylic sesquiterpene alcohol) are two prominent terpenes that show activity against *P. aeruginosa* MexAB-OprM efflux pump. Curcumin as a phenolic compound also blocks *P. aeruginosa* efflux pump MexAB-OprM and NorA, MdeA, TetK and MsrA efflux pumps from *S. aureus*. The steroid alkaloid conessine blocks MexAB-OprM efflux pump of *P. aeruginosa* [101]. Most of the substances screened as a potential EPI are metabolites from terrestrial vegetation. Marine ecosystem plants also hold potential for biodiscovery in this field. A recent study by Lu and colleagues reported that extract of brown seaweed *Laminaria japonica*, *Sargassum horneri* and that from red seaweeds *Gracilaria* sp. and *P. dentata* inhibit the efflux of ethidium bromide in multidrug resistant *E. coli*. Kam3-AcrB [102]. Looking at time-kill curves performed with the brown seaweed extract of *L. japonica, S. horneri*, and clarithromycin the results show a synergistic effect when added to the antibiotic but no effect on growth when added alone [102]. The number of viable cells exposed to a combination of clarithromycin and *L. japonica* extract (1/2 and 1/4 IC_50_) sharply decreased from 7 to 4.4 log CFU/mL after 12 h of incubation [102].

While considerable potential is associated with plant-derived EPI candidates, further synthetic development of these compounds is often hindered by the complexity of their chemical structure. Molecular modelling software may bring advances here by identifying specific structure/activity relationships allowing the targeting of specific synthetic modifications to natural compounds.

### 4.3. Marine Efflux Inhibitors

The marine environment is a vastly under-exploited area for the discovery of novel EPIs [103]. Mining the marine environment for such molecules could be beneficial, due to the range of diverse natural products, bioactive compounds already discovered from such environments via metagenomic-based technologies [104]. Furthermore, exposure to distinct stressors in the marine environment may potentially have evolutionary implications for the scope of efflux pump/inhibitor diversity, distribution and interplay therein. As mentioned above, QS and efflux pumps are intrinsically linked, whereby the modulation of one system can influence the functionality of the other. Microbes isolated from sea sponges have been found to encode novel QS inhibitors (QSIs) and could be a means of inhibiting MDR efflux pumps [105]. More directly, reversal of fluconazole resistance was also possible when combined with a marine sponge derived sulphated sterol isolated from *Topsentia* sp. [106]. Thus, marine environments, especially those with high microbial densities, e.g., sea sponge-associated, are worth exploring to combat AMR by exploring alternate/dual means of infection control (antibiotic and EPI, QSI and EPI, etc.).

### 4.4. Synthetic Efflux Inhibitors

Due to major advances in structure-function analysis, synthetic EPIs against specific microbial targets represent a promising means of infection control. Synthetic EPIs are generally built upon two scaffold molecules: quinolines and indoles [107]. These scaffolds are chosen as they lack intrinsic EPI activities. Only the modified molecule would have EPI activity. This targeting would need to be specific for the efflux pump and/or efflux pump family and for the species itself to avoid any secondary effects to neighboring microbes. This remains a challenge, as many of the natural substrates (aside from antibiotics) of efflux pumps remain to be identified. Promising advances in *Staphylococcal* control have been made through inhibition of the NorA efflux system, particularly where efflux inhibition has also been coupled with biofilm suppression. The 2-phenylquinoline scaffold has been particularly promising [85], synthetic derivatives of which were found to exhibit potent efflux inhibition of the NorA system [108]. Recently, the coupling of a scaffold hopping approach and a pharmacophore search led to a series of functionalized 2-arylquinazolines that exhibited strong synergism with ciprofloxacin. Importantly, these new efflux inhibitors exhibited a promising safety profile, with low cytotoxicity to human cells [109]. Phenylalanine-Arginine-β-Naphtylamide (PAβN) competitively targets three RND efflux pumps in *P. aeruginosa* (MexAB-OprM, MexCD-OprJ, and MexEF-OprN) of which it is also a substrate. Its broad substrate specificity enables it to potentiate the activity of a broad spectrum of antibiotics, including erythromycin and chloramphenicol [110]. Other efflux inhibitors include D13-9001, arylpiperazines, pyranopyridines, 2H-benzo[h]chromenes, NSC series compounds, TXA compounds, pyridylpiperazines (BDM compounds), reviewed recently by Compagne and colleagues [94]. IITR08027 was identified from a chemical library (*n* = 8000 synthetic molecules) screened for potentiators of ciprofloxacin, targeting *E. coli* and *A. baumannii* overespressing the MATE efflux pump AbeM [111]. MBX2319, a synthetic pyrazolopyridine, enhances the efficacy of ciprofloxacin, levofloxacin and piperacillin up to eight-fold against *E. coli* AB1157 [112]. Apparently, this latter EPI interacts with the AcrB pump, specifically targeting the hydrophobic trap [113,114].

### 4.5. Phage Steering

The co-evolution of bacteria and their viruses (i.e., bacteriophage) has been studied with some interest for many years. The close relationship between efflux pumps and phage whereby the latter uses the former as a receptor binding protein has been known for some time with examples including *P. aeruginosa* phage OMKO1 which binds to the M1 protein, *Vibrio cholerae* VP3, and *E. coli* U136B and TLS, both of which bind to TolC. Recent strategies for infection control have taken advantage of this co-evolution and receptor dependence, where bacteria are effectively forced to resist the selection pressure of either phage or antimicrobials [115]. In the case of *P. aeruginosa* OMKO1, downregulation of the OprM efflux protein led the bacteria to develop phage resistance with concomitant antimicrobial susceptibility [116]. However, such an approach requires prior knowledge of the evolutionary resistance pathways against both phage and antimicrobial for clinical success [117]. While the early research has provided some promising outcomes, a better understanding of phage ecology and evolution is needed before phage steering can be maximally exploited as an efflux-targeting AMR tool [50]. 

## 5. Efflux Pumps and Polymicrobial Communities 

Microbial life rarely exists in isolation in nature, yet traditional in vitro studies often focus on axenic cultures and settings. Instead, polymicrobial communities consist of various bacterial, archaeal, fungal, and viral species/variants. However, data pertaining to these mixed community studies are limited. In one study conducted utilizing mono-species wild-type vs. *acrAB-tolC* mutants and dual-species *E. coli*—*S. typhimurium* co-cultures, the presence of this MDR efflux pump in the community altered mutant growth rate when antagonized with antibiotics [118]. Thus, even variants/other microbes (“cheaters”) with lowered expression and/or no pump expression can benefit from efflux pump producers when antagonized with toxic substances. In fact, *E. coli-Salmonella* co-culture has been shown to increase biofilm formation and lead to an increased biocide resistance [118]. In some co-cultures, an increased efflux pump expression is observed, possibly as a defense mechanism against the other species, representing a shift from co-operative interaction to competitive exclusion [119]. However, in some instances, these “cheaters” do not benefit from producers, as producers exert a quorum control over efflux pumps when antagonized with certain antibiotics [120]. An example of this is where a *Burkholderia thailandensis* antibiotic, bactobolin, suppressed the emergence of QS-cheaters in *Chromobacterium violaceum* in co-culture leading to the *C. violaceum* population being more competitive in the co-culture model [120]. This demonstrates the diversity of possible interactions that can occur between different microbes under different environmental settings. 

As for endogenous waste export, it is likely alternative methods exist to extrude waste products from the cell within polymicrobial communities. Such endogenous waste products are recognized by other species and can influence efflux pump expression [121]. An example of this would be the recognition of indole (produced by *E. coli*) by *P. putida*, leading to the upregulation of TtgGHI efflux pump, as well as other genes involved in amino acid catabolism and iron homeostasis [121]. The production of indole and its subsequent detection by *P. putida* rescues TtgGHI-deficient strains when exposed to antibiotic antagonism. This would appear to be a secondary mechanism in natural ecosystems where the TtgABC efflux pump is the primary antibiotic extrusion system. Furthermore, as *P. putida* does not produce indole itself, this mechanism most likely evolved to reflect community level resistance, demonstrating the important ecological roles efflux pumps play in a polymicrobial setting. Studies involving more complex communities would be beneficial to better understand the true ecological role of efflux pumps. This is of particular importance given that the relationship between microbes is niche-specific and is constantly evolving in response to ever-changing environments. Furthermore, though most efflux pumps are present in the core genome, some are encoded on plasmids. As such, strains deficient in various efflux pumps could acquire efflux pumps (such as some MDR efflux pumps) via horizontal gene transfer (HGT). Thus, the polymicrobial perspective is a key component for the study of key physiological functions, though data and knowledge in this area are somewhat limited.

### Efflux Inhibition in the Polymicrobial Quorate

One major challenge to the clinical development of efflux inhibitor interventions is the realization that microbial communities are dynamic and undergo significant phenotypic and genotypic diversification, even at strain level. Therefore, functional redundancy, mutational adaptation, horizontal gene transfer, and target diversification all serve to challenge the efficacy and specificity of personalized interventions against pathogen colonization. Understanding the factors underpinning the complex and dynamic interactions that co-ordinate behavior in these mixed species communities is key to unlocking the potential of efflux intervention strategies to address multi-drug and pan-resistance of microbial pathogens. Studies have shown that drug efflux pumps were differentially expressed upon the co-culture of *P. aeruginosa* with streptococcal flora [122], with a role for quorum sensing and AI-2 signaling proposed. A role for the MexAB-OprM efflux pump has been proposed in growth antagonism of *P. aeruginosa* by *Enterococcus faecalis*, though the NalC-iron related mechanism of this modulation remains to be fully determined [123]. The recent finding that utilization of secondary metabolites produced by co-colonizing competitors underpins bacterial dominance suggests that efflux systems and metabolic flux will play an important role in shaping the dynamics of these communities [124]. Disrupting balance through off-target effects or unintended metabolic shifts could have consequences for the diversity and stability of the ‘healthy’ community in the ecosystem or niche in which the target pathogen resides.

One must also consider the influence of efflux inhibitors and efflux systems on the host-pathogen interaction, with several EPIs presenting activity against host targets [125].

## 6. Conclusions

Efflux pumps play critical key roles in the survival and proliferation of their encoding hosts. Efflux pumps can also benefit a polymicrobial community as a whole, especially when stressed with antimicrobial agents. As such, efflux pumps represent an antimicrobial resistance (AMR) mechanism in the current multi-drug resistance (MDR) era. A single efflux pump in a community can be enough to confer MDR. Though normally chromosomally encoded, efflux pumps can also be plasmid-encoded and acquired via horizontal gene transfer (HGT). Thus, continued surveillance of genotypic heterogeneity is needed to ensure the effectiveness of efflux inhibition is not lost to the onset of diversifying mutations.

Targeting resistance mechanisms is crucial to tackling MDR infections worldwide. Dual treatment of EPIs and antibiotics, or EPIs and other anti-virulence compounds, such as quorum-sensing inhibitors (QSIs) represents a promising infection control strategy. This is because efflux pumps are involved in key microbial cell processes, such as QS and signaling, metabolism, and other virulence factors, such as biofilm formation. However, many of these EPIs have fallen short due to cytotoxicity issues. Therefore, developing safe EPIs that selectively target a certain pathogen’s efflux pump(s) and/or its regulators is crucial here. In fact, anti-infective strategies targeting efflux pump regulators and its sensory systems may not carry the same risk of AMR development as EPIs on their own. However, it should be noted that, while early studies on anti-infective control often cited a lack of fitness cost, more recent studies have suggested otherwise [97]. Nevertheless, the cost is significantly less than that elicited by antibiotics, suggesting real potential in this promising research focus. Such EPIs and/or anti-infective strategies should also not alter the neighboring polymicrobial community profile. Future study needs to look at these issues under a polycellular lens, as well as keeping species and strain specificity in mind when studying microbial–microbial and microbial–host interactions Mining under-explored environments for natural EPIs or designing EPIs with a specific target in mind could enhance the development of combinatorial strategies, leading to clinically feasible and effective means of infection control.

## Figures and Tables

**Table 1:** The Major (Super)Families of Efflux Pumps in Bacteria.

Efflux Pump (Super) Family	Energy Source for Functionality	Membrane-Spanning Nature	Gram-Positive Example	Gram-Negative Example
ABC Superfamily	ATP Hydrolysis [1]	Forms a tri-partite complex in Gram-negative bacteria [1]	Sav1866—*Staphylococcus aureus* [11]	HlyB and MacB (TolC-dependent)—*Escherichia coli* [11]
RND Family	Proton Motive Force (PMF)—largely via respiration [1]	Forms a tri-partite complex [1]	Unique to Gram-negative bacteria [12]	MexAB-OprM in *Pseudomonas aeruginosa* [13]
MFS	Proton Motive Force (PMF)—largely via respiration [1]	Forms a tri-partite complex in Gram-negative bacteria	MdrM and MdrT in *Listeria monocytogenes*; NorA—*Staphylococcus aureus* [14]	EmrKY—*Shigella flexneri* [15]
SMR Family	Proton Motive Force (PMF)—largely via respiration [14]	Not known to form tri-partite complexes [9]	QacA/B—*Enterococcus faecalis* [14]	AbeS—*Acinetobacter baumannii* [14]
MATE Family	Proton Motive Force (PMF)—largely via respiration [14]	Not known to form tri-partite complexes [9]	MepA—*Staphylococcus aureus* [16]	NorM—*Vibrio cholerae* [14]
PACE Family	Proton Motive Force (PMF)—largely via respiration [17]	Not known to form tri-partite complexes [9]	Not known to be found in Gram-positive bacteria [17]	Acel—*Acinetobacter baumannii* [14]
AbgT Family	Proton Motive Force (PMF)—largely via respiration [18]	Forms a tri-partite complex in Gram—negative bacteria [18]	Homolog in *Streptomyces coelicolor* [18]	MtrF—*Neisseria gonorrhoeae* [18]

**Table 2:** Two-component system and one-component system regulators of RND efflux pumps in *P. aeruginosa*.

Environmental Cue(s)	Regulator(s)	Efflux Pump(s) Affected	Outcome(s)
Unclear	RocS2-RocA2 (TCS)	MexAB-OprM downregulated	Promotes biofilm formation
Antibiotic exposure (fluoroquinolone)	MexT (LTTR, OCS) Linked with the OprD porin repressor	MexEF-OprN activated	Antibiotic extrusion
Antibiotic exposure	MexL (TetR, OCS)	MexJK—downregulated by MexL	Antibiotic extrusion (multiple classes)
Antibiotic exposure, phenolic compounds	NalC (TetR, OCS)	Indirect repressor of MexAB-OprM (via ArmR)	
Antibiotic exposure	NalD (TetR, OCS)	Represses MexAB-OprM—secondary regulator	Antibiotic extrusion
Membrane- and envelope-damaging agents, e.g., antibiotics, biocides, dyes, solvents, etc.	NfxB (TetR, OCS)	Represses MexCD-OprJ	Antibiotic extrusion; antibiotic tolerance in biofilms
pH, temperature, oxidative stress, nutrient availability, toxins, etc.	MexR (MarR, OCS) linked with AmrR (controlled by the TetR repressor, NalC)	Primary activator MexAB-OprM	Antibiotic and other toxic substance extrusion; homeostasis
Ribosome stress, perhaps due to antibiotic (aminoglycoside) exposure—inducer unknown	MexZ (TetR, OCS) Most frequently mutated in Cystic Fibrosis patients	MexXY-OprM	Antibiotic extrusion
Envelope stress, membrane perturbation (colistin, polymyxin B)	ParR-ParS (TCS)	MexXY expressed MexEF-OprN upregulated via MexS (activator) activation	Antibiotic extrusion
	AmgR-AmgS (TCS)	MexXY expressed	Antibiotic extrusion
Heavy metal exposure	CzcR-CzcS (TCS)	CzcABC	Heavy metal extrusion
	CopR-CopS (TCS)	CzcABC	Heavy metal extrusion

## Data Availability

Not applicable.
